# Nutritional Risk in Patients with IBD: Results from a National Survey

**DOI:** 10.3390/nu18081261

**Published:** 2026-04-16

**Authors:** Camilla Fiorindi, Giulia Cei, Salvatore Leone, Enrica Previtali, Giorgia Burbui, Chiara Celli, Francesco Giudici

**Affiliations:** 1 Department of Health Science, University of Florence, 50100 Florence, Italy; 2 University of Florence, 50100 Florence, Italy; cei.giulia@libero.it (G.C.); giorgia.burbui@unifi.it (G.B.); celli.chiara@libero.it (C.C.); 3 AMICI ITALIA, Associazione Nazionale per le Malattie Infiammatorie Croniche dell’Intestino, 20125 Milan, Italy; salvo.leone@amiciitalia.net (S.L.); enrica.previtali@amiciitalia.net (E.P.); 4 Department of Experimental and Clinical Medicine, University of Florence, 50100 Florence, Italy; francesco.giudici@unifi.it

**Keywords:** inflammatory bowel disease, nutritional risk, MUST, malnutrition

## Abstract

**Background**: Malnutrition is a clinically relevant yet often under-recognized complication of inflammatory bowel diseases (IBD). Evidence from non-hospitalized patients remains limited. This study provides a descriptive analysis of indicators of nutritional risk among members of the Italian IBD patients’ association and explores the association with symptoms, clinical characteristics, and access to nutritional care. **Methods**: A cross-sectional survey was conducted on 705 IBD patients. The questionnaire incorporated all the items required to assess nutritional risk included in the Malnutrition Universal Screening Tool (MUST) and its association with other clinical parameters. **Results**: Overall, 43.0% of respondents were found to be at moderate or high risk of malnutrition based on the MUST. A low BMI was observed in 25.6% of patients with CD and 22.1% of those with UC. Additionally, unintentional weight loss (UWL) occurred in 24.5% and 23.7% of CD and UC patients, respectively. In total, 30% of patients reported restricting their diet because they were afraid certain foods would worsen their symptoms. Gastrointestinal symptoms were significantly associated with MUST ≥ 2. Only 21.7% of participants reported receiving nutritional counselling. A total of 67.3% of subjects already at high nutritional risk (MUST ≥ 2) have never received any dietary recommendations. **Conclusions**: In this community sample of IBD patients, a considerable proportion reported indicators compatible with nutritional risk. These findings are not generalized to the general IBD population due to the dissemination through a patient association, but highlight gaps in outpatient nutritional assessment and patient education. Structured, accessible nutritional support may help address these unmet needs.

## 1. Introduction

Malnutrition is a common condition in patients with inflammatory bowel disease (IBD) and often leads to altered body composition, with loss of lean mass and nutritional deficiencies [1]. The increased risk of a compromised nutritional status in IBD patients is due to changes in absorption and/or disease-related nutritional requirements, especially during active inflammation, often in combination with an unbalanced dietary intake [2,3]. According to the literature, a large proportion of patients with IBD are at risk of malnutrition, with estimates ranging from 16% to 75%, depending on the parameters used for nutritional assessment [3]. The etiology of malnutrition is multifactorial and results from a combination of factors such as the inflammatory state, clinical complications of the disease (e.g., fistulas, strictures, abscesses), and previous surgical resections, which expose patients to malabsorption and nutrient loss [1,4]. A compromised nutritional status is therefore a consequence of the disease, although it can also further affect its course. This can lead to a vicious cycle where poor nutritional status and intestinal inflammation influence each other [5]. Malnutrition is also known to affect the composition and activity of the gut microbiota, potentially impacting disease progression [6]. For these reasons, it is essential to promptly identify IBD patients at nutritional risk [5,7,8]. The European Society for Clinical Nutrition and Metabolism (ESPEN) guidelines on clinical nutrition in IBD emphasize the increased risk of malnutrition; therefore, they must undergo nutritional screening at the time of diagnosis and subsequently on a regular basis [5]. A recent online survey conducted across various Italian IBD centres has highlighted that nutritional care for IBD patients is far from satisfactory [9]. Educational and structural interventions are urgently needed to improve the assessment and treatment of malnutrition in everyday clinical practice [9]. The aim of our study was to determine the prevalence of malnutrition risk by conducting an online survey of members of a national IBD patients’ association. The survey was designed to include a large number of subjects and assess the extent of the nutritional risk. Additionally, we aimed to collect data on key factors influencing nutritional risk, such as disease characteristics, gastrointestinal symptoms, and access to nutritional education, which could also be collected through the online survey.

## 2. Materials and Methods

### 2.1. Study Design and Population

A cross-sectional online survey was distributed by Italian IBD patients’ association A.M.I.C.I. Italia, which has 2993 registered members. The survey was proposed to all association members via newsletters, email and social media platforms. Patient recruitment for the survey occurred within the timeframe of June to August 2024.

Inclusion criteria:

- Age ≥ 18 years;
- Self-reported diagnosis of Crohn’s disease (CD), ulcerative colitis (UC), or indeterminate colitis;
- Not hospitalized at the time of survey completion;
- Provision of electronic informed consent.

Exclusion criteria:

- Exclusive enteral or parenteral nutrition;
- Duplicate responses;
- Incomplete questionnaires.

### 2.2. Questionnaire Development

The 58-item questionnaire required approximately 10–12 min to complete. It included:

- Age, Gender, Region of residence, Education level, Comorbidities, Smoking habits, Level of physical activity;
- Self-reported weight, height, and weight 6 months prior;
- Body Mass Index (BMI);
- Gastrointestinal symptoms and appetite reduction;
- Recent inflammation markers (<2 weeks) as *C*-Reactive Protein;
- Disease history, medical therapy, and prior surgeries;
- Use of vitamins, minerals and oral nutritional supplements (ONS);
- Access to dietary counselling.

Mandatory fields were applied to essential items; skip logic was used for conditional questions.

### 2.3. Nutritional Risk Assessment

The ‘Malnutrition Universal Screening Tool’ (MUST) is a validated nutrition screening tool developed by BAPEN to help identify adults at risk of undernutrition and the need for dietary advice. MUST is the most common nutritional screening tool used by healthcare professionals. Studies indicate that patients can effectively screen themselves, as the tool is easy to understand and complete [10,11]. Research has demonstrated a substantial to almost perfect an agreement between patient self-reported MUST scores and those performed by healthcare professionals [12].

The MUST involves a systematic assessment based on three key parameters. These parameters are combined to determine an overall risk score and guide a management plan. MUST was used exclusively as a screening tool, not a diagnostic measure.

1. BMI: This was calculated using the patient’s self-reported height and weight.
2. Unintentional weight loss (UWL): This was determined as the percentage of UWL over the past three to six months. For this reason, patients were asked to report their weight from six months prior.
3. Acute Disease Effect: This element assessed the patient’s self-reported disease activity combined with a reduced/no nutritional intake for more than five days. Specifically, this item was determined from self-reported data by asking participants whether they had consistently reduced their daily food intake over the past week (defined as a reduction of less than 50% of their usual portions).

Medium or high risk of malnutrition was considered in patients with MUST score of 1 or ≥2, respectively.

### 2.4. Statistical Analysis

A descriptive analysis was carried out to observe the characteristics of the sample, focusing on data related to nutritional risk assessment using the MUST. Categorical variables were analyzed using the Chi-Square Test, along with Cramér’s V to measure the strength of association, with statistical significance set at *p* < 0.05. Fisher’s Exact Test was used as a confirmation when the Chi-Square Test was not valid due to cell frequencies < 5 in the contingency table. Given the exploratory nature of the study, no formal adjustment for multiple comparisons was applied. The analyses were primarily aimed at identifying potential associations rather than testing a limited number of predefined hypotheses; therefore, *p*-values should be interpreted with caution.

To further investigate factors independently associated with nutritional risk, a multivariate logistic regression model was performed using MUST ≥ 2 as the dependent variable. Variables were selected a priori based on clinical relevance and the existing literature, including demographic and disease-related characteristics. Only patients with a confirmed diagnosis of Crohn’s disease or ulcerative colitis were included in the regression analysis, while those with indeterminate colitis were excluded due to their small number. Results were reported as odds ratios (ORs) with 95% confidence intervals (CIs).

### 2.5. Data Quality

Automatic controls checked for duplicated responses using IP address, browser hash, and timestamp. Only complete questionnaires were analyzed.

### 2.6. Ethical Consideration

Due to the lack of direct interaction between the research team and the participants, informed consent was obtained remotely; all patients provided their consent via an electronic form approved by the AMICI ITALIA committee.

## 3. Results

### 3.1. Participants

**Figure 1:**
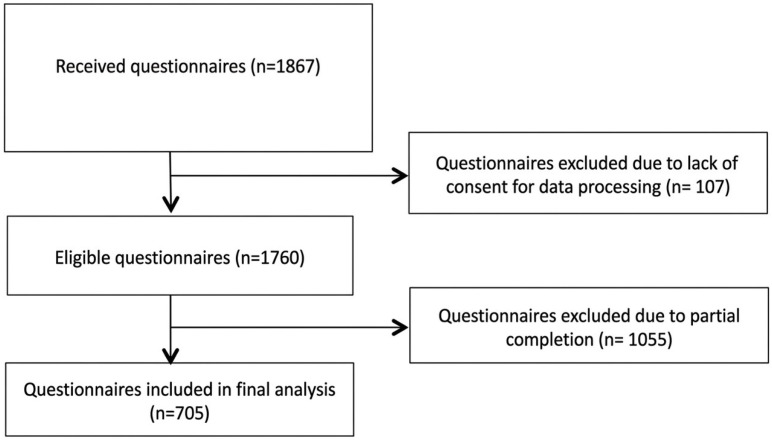
Flowchart of questionnaires’ assessment for eligibility.

Overall, the collected sample consists of 1867 questionnaires. In total, 1055 were not fully completed and 107 patients did not give the consent for data processing and they were excluded. Therefore 705 patients’ questionnaires were included, as shown in Figure 1.

The cohort comprised 327 patients (46.4%) with CD, 358 (50.8%) with UC, and 20 (2.8%) with indeterminate colitis. The sex distribution was essentially overlapping, with 55.5% of respondents being female and the remaining 44.5% male. Disease duration was less than 10 years in 31.9% of respondents, between 10 and 20 years in 28.8%, between 20 and 30 years in 18.2%, between 30 and 40 years in 15.2%, and greater than 40 years in 6% of subjects. Regarding self-reported disease activity, 73.9% of respondents declared being in remission, while 26.1% reported active disease. Of these, only 1% stated recent hospitalization for active disease. Patients reporting a history of abdominal surgery for IBD were 32.9% of the sample, including 58.1% of those with CD and 10.6% of those with UC. ONS consumption was reported by 12.3% of respondents, specifically 13.1% of those with CD and 10.6% of those with UC. Among these, 71.2% reported using CD-specific powdered nutritional supplements, while 28.8% reported taking high-calorie, high-protein liquid supplements (125–200 mL/brick). Regarding vitamin supplementation, 52.1% of the sample reported intake, whereas 34.8% declared the use of mineral supplementation. Physical activity was performed by 55.5% of the sample, specifically 56.3% of subjects with CD and 54.7% with UC. The most reported types of physical activity were attending the gym (classes, weight training) 3 to 5 times per week (21.5% of the sample) and walking for at least 30 min 3 to 5 times per week (15.6% of the sample). A total of 37.7% of the respondents reported at least one gastrointestinal (GI) symptom, including 38.2% of those with CD and 35.8% with UC. Reported GI symptoms included diarrhea 39.2%, abdominal pain 21.2%, abdominal bloating 36.9% and nausea 2.7%. Finally, 21.7% of the sample reported having received dietary recommendations, particularly 56.2% of those with CD and 39.2% of those with UC. Subjects with CD were more likely to undergo abdominal surgery, use vitamin supplements (particularly B12 and B9), use multimineral supplements, and report greater access to dietitian consultation than subjects with UC (Table 1).

### 3.2. Nutritional Screening According to MUST

The assessment based on the MUST showed a high risk of malnutrition in 30.8% of subjects, and a moderate risk of malnutrition in 12.2% of respondents, totaling 43% of subjects at moderate or high risk (Table 2).

**Figure 2:**
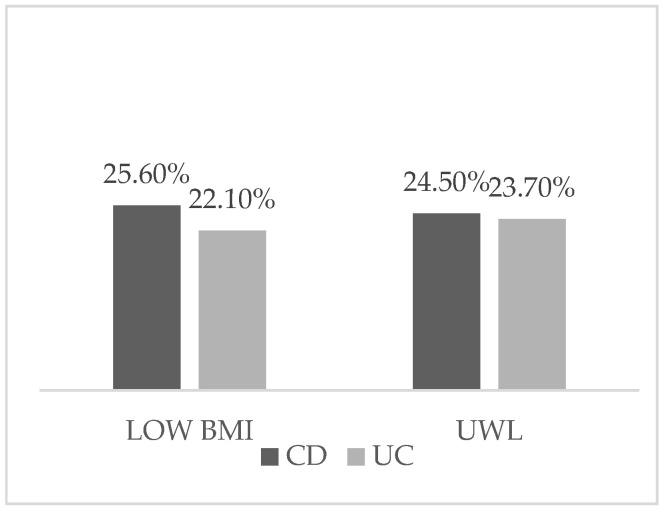
*Prevalence of low BMI and UWL in CD and UC subjects.* CD: Crohn’s disease (CD); UC: ulcerative colitis; BMI: Body Mass Index; UWL: unintentional weight loss.

A low BMI was observed in 25.6% of patients with CD and 22.1% of those with UC. Low BMI is defined as <20 kg/m^2^ for subjects aged <70 years and <22 kg/m^2^ for subjects aged >70 years. Additionally, unintentional weight loss (UWL) occurred in 24.5% and 23.7% of CD and UC patients, respectively (Figure 2).

### 3.3. Correlation Between Nutritional Risk and Clinical Characteristics, Lifestyle and Access to Nutritional Care

The associations between nutritional risk and clinical factors including disease activity, time since diagnosis, history of abdominal surgery, and disease location were evaluated, but no statistically significant relationships emerged (Table 3). Analyzing the correlation between the presence of at least one gastrointestinal symptom and nutritional risk according to MUST, a highly significant association emerged (p 0.001); therefore, those who present at least one gastrointestinal symptom could be more exposed to the risk of malnutrition. Regarding the correlation between ONS intake and nutritional risk according to the MUST, a significant association emerged (*p* = 0.014). Among the respondents classified as being at nutritional risk according to MUST criteria, 35.53% did not receive dietary recommendations; of these, 11.75% were at moderate risk and 23.78% were at high risk.

In our sample, 29.9% reported restricting their diet due to concerns about worsening gastrointestinal symptoms. Among these individuals, only 41.7% received professional dietary recommendations. Specifically, dietary assistance was provided by a gastroenterologist in 28.4% of cases or by a physician nutritionist or dietitian in 39.8% of cases. Of the 58.3% of participants who restricted their diet due to fear of symptoms without professional guidance, 64.2% sought nutritional information independently, with 36.7% relying on the internet as their primary source. Analysis of the association between the elimination of specific foods and beverages and independent information-seeking behaviour revealed a significant correlation (*p* < 0.05), indicating that individuals who exclude foods or drinks from their diet are more likely to seek nutritional information autonomously. Participants who engaged in physical activity demonstrated a slightly lower mean BMI. The mean BMI among individuals who were physically inactive was 23.94 kg/m^2^ (SD ± 4.97), compared with 22.67 kg/m^2^ (SD ± 3.62) among those who were active (p 0.001).

### 3.4. Multivariate Analysis of Factors Associated with Nutritional Risk (MUST ≥ 2)

A multivariate logistic regression analysis was performed to identify factors independently associated with nutritional risk (MUST ≥ 2).

The presence of at least one gastrointestinal symptom was significantly associated with an increased likelihood of being at nutritional risk (OR 1.65, 95% CI 1.13–2.43, *p* = 0.010).

Female sex was also independently associated with a higher risk of malnutrition (OR 2.52, 95% CI 1.81–3.54, p 0.001). In addition, patients reporting access to dietary counselling showed a significantly higher probability of being at nutritional risk (OR 2.12, 95% CI 1.42–3.19, p 0.001).

Age showed a borderline inverse association with nutritional risk (OR 0.99, 95% CI 0.97–1.00, *p* = 0.050), suggesting a slightly lower risk with increasing age. No statistically significant associations were observed for disease duration, disease type (Crohn’s disease vs. ulcerative colitis), self-reported disease activity or previous abdominal surgery (Table 4).

## 4. Discussion

This study represents the largest online survey conducted in Italy to date on the risk of malnutrition in non-hospitalized patients with IBD. Our findings show that a significant proportion of patients are at nutritional risk. There are some studies in the literature on patients with IBD where nutritional risk was assessed using self-administrated MUST screening tools, but not in electronic format. Recent studies [10,11] have evaluated the feasibility and validity of a patient-administered MUST in an IBD outpatient clinic. The results of these studies demonstrate the excellent efficacy of self-administered nutritional screening tests, suggesting that their implementation could improve the nutritional management of patients with IBD. In our multivariate analysis, the presence of gastrointestinal symptoms emerged as an independent factor associated with nutritional risk. Patients reporting at least one GI symptom were significantly at risk of malnutrition. This finding is consistent with current evidence, as symptoms such as abdominal pain, diarrhea, bloating, and nausea can directly impair dietary intake and reflect ongoing inflammatory activity. These results support the importance of integrating symptom assessment with nutritional screening, rather than relying solely on anthropometric measures.

The presence of specific gastrointestinal symptoms, such as nausea, vomiting, bloating, abdominal pain and decreased appetite, have been incorporated as supportive indicators into GLIM consensus, as they can indirectly reveal the presence of etiological criteria [13]. Reduced appetite and decreased oral food intake are key contributors to malnutrition risk in patients affected by IBD. Two main mechanisms are reported: the first is connected to the disease itself, as patients avoid eating due to symptoms during disease flare [14,15]; the second involves voluntary dietary restriction aimed at preventing symptom exacerbation, which may further compromise nutritional status. Interestingly, female sex was independently associated with a higher risk of malnutrition. This finding may reflect differences in disease perception, dietary behaviours, or reporting patterns, although further studies are needed to clarify the underlying mechanisms.

Another notable finding is the association between access to dietary counselling and increased nutritional risk. This result should be interpreted with caution, as it likely reflects reverse causation: patients at higher nutritional risk are more likely to be referred for dietary consultation. This highlights the importance of timely nutritional assessment and referral, particularly in high-risk individuals.

From a clinical management perspective, a major concern is that only 21.7% of patients reported receiving dietary recommendations from a healthcare professional, and more than one-third of those at nutritional risk had not received any nutritional guidance. Furthermore, many patients reported modifying their diets independently, often by restricting foods to avoid worsening symptoms, which could inadvertently aggravate their nutritional status. As reported by Casanova et al. avoidance of some foods during flares is associated with higher risk of malnutrition [16].

These observations reflect the widespread lack of nutritional education commonly observed in patients with IBD, as also reported in the study by Fiorindi et al. [17], in which a large proportion of subjects exhibited inadequate food literacy, associated with poorer health outcomes and reduced quality of life.

### Strengths and Limitations

This study has some limitations. The self-reported nature of the questionnaire may introduce recall and reporting biases, particularly regarding weight, BMI, and bio-chemical markers. Additionally, dissemination through a patient association may have selected for individuals who are more health-conscious compared to the general IBD population, introducing a self-selection (volunteer) bias. Given the exploratory nature of the study, no formal adjustment for multiple comparisons was applied, and results should therefore be interpreted with caution. However, to strengthen the robustness of our findings, a multivariate logistic regression analysis was performed to account for potential confounding factors.

## 5. Conclusions

In this community sample, a sizable proportion of AMICI members reported indicators compatible with nutritional risk. Although not generalizable to the broader IBD population, the findings underscore the need to strengthen outpatient nutritional screening, patient education and access to dietary counselling. Further prospective studies are warranted to better define causal relationships and to further investigate determinants of nutritional risk in patients with IBD.

## Figures and Tables

**Table 1:** Characteristics of IBD subjects.

	*IBD*	*CD*	*UC*	*p* *
n° of subjects	705	327 (46.4%)	358 (50.8%)	
Age, mean (SD)	52.18 (±14.29)	51.74 (±14.34)	52.58 (±14.24)	0.440
Male, n°	305 (44.5%)	149 (45.6%)	156 (43.6%)	0.600
Disease duration, years, mean (SD)	18.12 (±12.63)	19.21 (±12.6)	17.14 (±12.6)	0.032
Previous surgery, n° of subjects	232 (32.9%)	190 (58.1%)	38 (10.6%)	0.001
Number of abdominal surgeries for IBD, mean (SD)	2.37 (±1.66)	2.35 (±1.75)	2.44 (±1.17)	0.378
Self-reported active disease, n°	184 (26.1%)	94 (28.7%)	82 (22.9%)	0.097
ONS intake, n° of subjects	87 (12.3%)	43 (13.1%)	38 (10.6%)	0.364
Intake of vitamins, n° of subjects	367 (52.1%)	193 (59.0%)	166 (46.4%)	0.001
Vitamin D3, n° of subjects	227 (62%)	110 (57.0%)	111 (67.0%)	0.513
Vitamin B12, n° of subjects	123 (34%)	81 (42.0%)	41 (25.0%)	0.001
Vitamin B9, n° of subjects	107 (29%)	66 (34.0%)	38 (23.0%)	0.001
Vitamin B complex, n° of subjects	77 (21%)	37 (19.0%)	39 (23.0%)	0.957
Multivitamin supplement, n° of subjects	112 (31%)	60 (31%)	49 (30%)	0.118
Intake of minerals, n° of subjects	245 (34.8%)	125 (38.2%)	111 (31.0%)	0.057
Iron. n° of subjects	81 (33%)	44 (35.0%)	31 (28.0%)	0.059
Zinc. n° of subjects	41 (17%)	16 (13.0%)	22 (20.0%)	0.584
Selenium. n° of subjects	14 (6%)	5 (4.0%)	8 (7.0%)	0.692
Calcium. n° of subjects	53 (22%)	29 (23.0%)	23 (21.0%)	0.288
Potassium, n° of subjects	67 (27%)	28 (22.0%)	35 (32.0%)	0.677
Magnesium, n° of subjects	99 (40%)	43 (34.0%)	52 (47.0%)	0.682
Multimineral supplement, n° of subjects	367 (150%)	71 (57.0%)	51 (46.0%)	0.014
Practice of physical activity, n° of subjects	391 (55.5%)	184 (56.3%)	196 (54.7%)	0.747
Presence of at least one GI symptom, n° of subjects	263 (37.7%)	125 (38.2%)	128 (35.8%)	0.555
Access to dietary consultation, n° of subjects	153 (21.7%)	86 (26.2%)	60 (16.7%)	0.001

* *p*-values CD vs. UC (due to the very low number (<=5), the percentages relating to indeterminate colitis were not included). IBD: inflammatory bowel disease; CD: Crohn’s disease (CD); UC: ulcerative colitis; GI: gastrointestinal; ONS: oral nutritional supplements.

**Table 2:** Prevalence of nutritional risk according to MUST score.

	*IBD*	*CD*	*UC*	*p* *
Low risk of malnutrition (MUST 0), n*°*	402 (57.0%)	182 (55.7%)	209 (58.4%)	0.052
Medium risk of malnutrition (MUST 1), n*°*	86 (12.2%)	45 (13.8%)	38 (10.6%)	0.253
High risk of malnutrition (MUST ≥ 2), n*°*	217 (30.8%)	100 (30.6%)	111 (31.0%)	0.970

* *p*-values CD vs. UC. IBD: inflammatory bowel disease; CD: Crohn’s disease (CD); UC: ulcerative colitis; MUST: Malnutrition Universal Screening Tool.

**Table 3:** Characteristics of IBD subjects according to nutritional risk. * *p*-values CD vs. UC (due to the very low number (<=5), the percentages relating to indeterminate colitis were not included).

	*MUST* 0	*MUST* 1	*MUST* ≥ 2	*p* *
n° of subjects	402 (57%)	86 (12.2%)	217 (30.8%)	
Disease duration, years, mean (SD)	18.8 (±12.8)	16.7 (±12.3)	16.9 (±12.4)	0.042
Previous surgery, n° of subjects	132 (32.8%)	30 (34.9%)	71 (32.7%)	0.916
Number of abdominal surgeries for IBD, mean (SD)	2.51 (±2.08)	2.17 (±1.84)	2.96 (±2.49)	0.391
Self-reported active disease, n° of subjects	92 (22.9%)	24 (27.9%)	68 (31.3%)	0.067
ONS intake, n° of subjects	38 (9.5%)	11 (12.8%)	38 (17.5%)	0.014
Intake of vitamins, n° of subjects	196 (48.8%)	51 (59.3%)	120 (55.3%)	0.106
Vitamin D3, n° of subjects	119 (60.7%)	33 (67.4%)	75 (62.5%)	0.858
Vitamin B12, n° of subjects	69 (35.2%)	18 (35.3%)	36 (30%)	0.609
Vitamin B9, n° of subjects	53 (27%)	15 (29.4%)	39 (32.5%)	0.584
Vitamin B complex, n° of subjects	41 (20.9%)	10 (19.6%)	26 (21.7%)	0.954
Multivitamin supplement, n° of subjects	56 (30.1%)	11 (21.6%)	45 (37.5%)	0.080
Intake of minerals, n° of subjects	117 (29.1%)	37 (43%)	91 (41.9%)	0.001
Iron, n° of subjects	36 (30.8%)	8 (21.6%)	35 (38.5%)	0.162
Zinc, n° of subjects	14 (12%)	7 (18.9%)	20 (22%)	0.147
Selenium, n° of subjects	4 (3.4%)	1 (2.7%)	9 (9.9%)	0.094
Calcium, n° of subjects	24 (20.5%)	8 (21.6%)	20 (22%)	0.965
Potassium, n° of subjects	32 (27.4%)	8 (21.6%)	27 (29.7%)	0.651
Magnesium, n° of subjects	42 (35.9%)	21 (56.8%)	36 (42.9%)	0.077
Multimineral supplement, n° of subjects	57 (48.7%)	16 (43.2%)	48 (52.7%)	0.609
Practice of physical activity, n° of subjects	230 (57.2%)	53 (61.6%)	108 (49.8%)	0.096
Presence of at least one GI symptom, n° of subjects	122 (30.3%)	36 (41.9%)	105 (48.4%)	0.001
Access to dietary consultation, n° of subjects	58 (14.4%)	24 (27.9%)	71 (32.7%)	0.001

MUST: Malnutrition Universal Screening Tool; GI: gastrointestinal; ONS: oral nutritional supplements.

**Table 4:** Multivariate logistic regression analysis of factors associated with nutritional risk (MUST ≥ 2). Odds ratios (ORs) and 95% confidence intervals (CIs) are reported. Reference categories are indicated in parentheses.

*Variable*	*OR*	*CI_Low* (2.5%)	*CI_High* (97.5%)	*p* *
Age	0.987	0.974	1.000	0.050
Sex	2.523	1.807	3.539	0.001
Disease duration	1.004	0.988	1.020	0.607
Crohn’s disease	0.948	0.650	1.382	0.780
Self-reported active disease	1.027	0.676	1.566	0.902
Presence of at least one GI symptom	1.654	1.130	2.426	0.010
Previous surgery	0.945	0.614	1.451	0.796
Access to dietary consultation	2.124	1.423	3.189	0.001

* *p*-values (due to the very low number (<=5), the percentages relating to indeterminate colitis were not included); GI: gastrointestinal.

## Data Availability

The data that support the findings of this study are available from the corresponding author upon reasonable request due to privacy reasons.

## Abbreviations

The following abbreviations are used in this manuscript:

IBD	Inflammatory Bowel Diseases
CD	Crohn Disease
UC	Ulcerative Colitis
MUST	Malnutrition Universal Screening Tool
GLIM	Global Leadership Initiative on Malnutrition
BMI	Body Mass Index
UWL	Unintentional weight loss
GI	Gastrointestinal
ONS	Oral Nutritional Supplement
